# Factors affecting compliance and non-compliance among patients with tuberculosis in Egypt: a descriptive correlational study

**DOI:** 10.7586/jkbns.25.070

**Published:** 2026-02-27

**Authors:** Hanan Mohammed Mohammed

**Affiliations:** 1 Medical-Surgical Nursing Department, Faculty of Nursing, Al-Baha University, Saudi Arabia; 2 Medical-Surgical Nursing Department, Faculty of Nursing, Ain Shams University, Egypt

**Keywords:** Antitubercular agents, Egypt, Patient compliance, Tuberculosis

## Abstract

**Purpose:**

This study aimed to assess factors associated with compliance and non-compliance among patients with tuberculosis (TB).

**Methods:**

A descriptive correlational study was conducted at the outpatient clinics of Abbassia Chest Hospital in Cairo, Egypt. A sample of 80 patients with TB who had been prescribed treatment regimens was recruited. Patients were interviewed using a structured form with fixed response options to identify factors associated with treatment compliance or non-compliance.

**Results:**

A significantly higher proportion of non-compliant patients (72.5%) experienced severe symptoms compared with patients in the compliant group (30.0%). Furthermore, most compliant patients (82.5%) demonstrated satisfactory overall knowledge of pulmonary TB, whereas only about half (52.5%) of patients in the non-compliant group did. This difference in knowledge level was statistically significant and was associated with treatment adherence. Among compliant patients, knowledge levels were also significantly influenced by age, sex, education, and employment status, a pattern that was not observed in the non-compliant group.

**Conclusion:**

Key factors associated with non-compliance included insufficient income to support treatment, lack of home support for treatment regulation, and decreased motivation to improve health. These findings underscore the need for targeted patient education and counselling programs to increase disease awareness and improve treatment adherence among patients with TB.

## INTRODUCTION

Tuberculosis (TB) remains a major global public health threat, but its burden is not evenly distributed, with specific challenges emerging at the regional and national levels. In Egypt, TB persists as a significant public health issue, even amidst global improvements in diagnosis and treatment. The World Health Organization (WHO) reports that Egypt has an estimated TB incidence of 11 cases for every 100,000 people, with treatment success rates below the global goal of 90% with an estimated mortality rate (including TB among HIV-positive people) of 3.1 deaths per 100,000 population in 2022. This translates to approximately 3,300 deaths annually, positioning Egypt's TB mortality burden lower than the global average and reflecting the impact of the country's sustained national TB control efforts [1]. Despite this achievement, TB remains a persistent public health threat, and these figures underscore the critical need to address drivers of mortality, such as treatment non-adherence and the rise of drug-resistant strains, to accelerate the decline in TB-related deaths [2]. Tackling these issues is crucial for improving treatment outcomes and lowering the TB burden in Egypt [3].

In Egypt, the sociocultural landscape significantly exacerbates the risk of TB treatment non-compliance, a problem whose scale is reflected in national treatment success rates that often fall short of WHO targets. Deeply entrenched stigma, fueled by misconceptions that TB is a shameful disease or a divine punishment, drives patients, particularly women, to conceal their diagnosis and avoid clinic visits to prevent social ostracization and protect family honor [4]. This is compounded by widespread health illiteracy and cultural beliefs that prioritize traditional remedies over biomedical treatment, leading patients to abandon therapy once initial symptoms subside. Furthermore, the substantial economic burden of treatment, including lost wages from frequent clinic visits and costs of transportation and nutritious food—forces many low-income earners to choose between their health and their livelihood [5]. The consequences of this non-adherence are severe at both the individual and public health levels, leading to increased drug resistance, higher mortality, and continued community transmission, as untreated or partially treated patients, even those initially sputum-negative, remain a reservoir for the bacillus, perpetuating a cycle of disease and poverty that poses a major obstacle to national TB control efforts [6].

Beyond individual financial hardship, structural weaknesses within the healthcare system itself also play a big role in non-compliance. Long distances to treatment centers, frequent drug shortages, and lack of patient counseling are key challenges. Egypt’s centralized TB control program, though effective in urban settings, has trouble reaching rural areas, where patients often experience delays in diagnosis and fragmented care. Moreover, the absence of patient-centered approaches, such as limited health education and poor communication between providers and patients, worsens non-adherence [7]. Improving decentralized care, expanding telemedicine options, and better training for healthcare workers could help close these gaps [8]. Compounding these systemic and economic factors are patient-related issues, such as lack of knowledge, stigma, and other health conditions, which make it even harder for patients to stick to their treatment. Many TB patients in Egypt are not fully aware of how serious the disease is or the risks of incomplete treatment [9].

Selecting sputum-negative TB patients as the study population is twofold, addressing a critical gap in both clinical management and public health strategy. Firstly, sputum-negative TB often presents with milder or atypical symptoms, leading to delayed diagnosis and a potential underestimation of disease severity by both patients and clinicians; this diagnostic ambiguity can foster complacency, making this group particularly vulnerable to non-adherence as they may feel less ill and therefore less motivated to complete the lengthy treatment regimen [10]. Secondly, from a public health perspective, while sputum-negative patients are considered less infectious, they still contribute significantly to community transmission, especially if their treatment is interrupted, which can lead to relapses and potential progression to contagious sputum-positive disease [11]. Therefore, understanding and addressing the unique adherence challenges in this understudied but epidemiologically important patient group is essential for developing targeted interventions that not only improve individual outcomes but also ultimately disrupt the chain of TB transmission within the community.

Finally, the framework of the current study is contextualized within Egypt's unique socioeconomic and health-system environment, acknowledging that external factors like poverty, limited social support, and healthcare access are not merely background variables but active determinants that directly shape the individual perceptions and self-efficacy. This integrated approach allows the study to move beyond a simple description of factors and toward an explanatory model of how cognitive, behavioral, and environmental dynamics interact to drive compliance or non-compliance [12]. In Egypt, where alternative medicine and self-medication are widespread, some patients stop taking prescribed TB medications and turn to unproven remedies instead [10]. Programs to monitor drug safety and better patient counseling on managing side effects are crucial for improving adherence [13].

Understanding treatment adherence dynamics in Egypt requires a comprehensive approach that specifically includes sputum-negative TB patients, as their unique diagnostic and psychosocial challenges create distinct barriers to care [3]. This focus is critical because, unlike their smear-positive counterparts, sputum-negative patients often face significant diagnostic delays in the Egyptian healthcare system, which can erode trust in the medical establishment and de-motivate patients from the outset of their long and arduous treatment regimen. Furthermore, the absence of confirmatory bacteriological evidence can exacerbate social stigma, thereby weakening the crucial social support network that is a known pillar of treatment adherence. Consequently, excluding this significant patient subgroup would yield an incomplete and potentially biased understanding of adherence factors, as the drivers of non-compliance for them may differ qualitatively [5,6].

Therefore, a thorough investigation of the factors affecting compliance and non-compliance must intentionally integrate the sputum-negative experience to ensure that future supportive interventions in Egypt are equitable, inclusive, and effectively tailored to the needs of the entire TB patient population. This study aimed to assess the factors affecting compliance and non-compliance among patients with TB.

## METHODS

### 1. Study design

A descriptive correlational study design was utilized in carrying out this study. Additionally, the study aimed to assess factors affecting compliance and non-compliance among patients with TB.

### 2. Study setting

The current study was conducted at outpatient chest clinic at Abbassia Chest Hospital (ACH) in Cairo, Egypt.

#### Participants

Purposive sampling for patients with TB attending the study setting and who is identified as sputum negative for TB was eligible for inclusion in the study sample. The inclusion criteria were age more than 18 years, male or female, and accepting to participate in the study. The study excluded patients with associated pulmonary diseases such as lung cancer, chronic obstructive pulmonary disease, bronchial asthma, and infected or with chronic pulmonary TB were excluded as well to minimize confounding clinical variables that could independently affect treatment adherence.

The sampling frame (the patient roster) was used as a pool from which to purposefully identify patients who met the strict operational definitions for compliance and non-compliance. A stratified selection process was used to purposefully enroll a balanced number of participants into two key groups: "compliant" and "non-compliant." Patients were assigned to these groups based on strict, pre-defined operational definitions of compliance (A positive behavior and a state in which the patient follows the therapeutic regimen, taking ≥ 90% of prescribed doses that are required for management of his/her illness) and non-compliance (A state in which the patient does not follow the therapeutic regimen taking < 90% of doses or having treatment interruptions that is required for the management of his/her illness), in other words, the patient does not adhere to drugs intake and has no regular attendance to outpatient clinic for follow-up. [1,14] This deliberate stratification was not random; it was designed to ensure that sufficient participants from each critical category were included. This method guaranteed a robust sample size for a powerful comparative analysis of the factors differentiating patients with strong adherence from those struggling with it.

For this study, a patient was considered non-compliant if he/she failed to take seven doses of drugs, either sporadic or continuous, and/or if he/she is a defaulter patient, i.e. absent from follow-up more than two months.

The sample size was calculated based on the following formula [15].

The formula for the sample size per group (n) is:

n = [Zα/2√(2P(1-P)) + Zβ√(P1(1-P1) + P2(1-P2))]^2^ / (P1 - P2)^2^

Description:

The analysis was based on the following parameters:

• Statistical Power (1-β): 80% (Zβ = 0.84)

• Significance Level (α): 5% (Zα/2 = 1.96 for a two-sided test)

• P1 (Proportion in compliant group): 0.80 (80% anticipated to have satisfactory knowledge)

• P2 (Proportion in non-compliant group): 0.50 (50% anticipated to have satisfactory knowledge)

• Effect Size (P1 - P2): 0.30 (A 30 percentage point difference, considered clinically significant)

Step-by-step calculation

1. Zα/2√(2P(1-P)) = 1.96 * √(2 * 0.65 * 0.35) = 1.96 * √(0.455) = 1.96 * 0.674 = 1.321

2. Zβ√(P1(1-P1) + P2(1-P2)) = 0.84 * √( (0.80*0.20) + (0.50*0.50) )

= 0.84 * √(0.16 + 0.25) = 0.84 * √(0.41) = 0.84 * 0.640 = 0.538

3. Sum of (1) and (2) = 1.321 + 0.538 = 1.859

4. (P1 - P2)^2^ = (0.30)^2^ = 0.09

5. n per group = (1.859)^2^ / 0.09 = 3.456 / 0.09 = 38.4

The calculation indicated that a minimum of 38.4 participants per group was required. To account for potential attrition and ensure adequate power, this number was rounded up to 40 participants per group. Thus, the total target sample size for the study was 80 patients. To ensure statistical adequacy, a post-hoc power analysis was conducted using G*Power software (Version 3.1). With a fixed sample size of 80 participants (40 per group), an alpha (α) level of 0.05, and an effect size (Cohen's w) of 0.35 (based on preliminary data indicating a medium effect), the analysis determined the study had a statistical power (1-β) of 78% for a Chi-square test of independence. This power level exceeds the conventional threshold of 80% for detecting a medium effect size, thereby confirming that the sample size was adequate to identify statistically significant associations between patient groups and the primary outcome variables.

### 3. Instruments

A structured interview sheet was designed and used to assess the factors leading to non-compliance toward therapeutic regimen. It was developed in simple Arabic language. Structured interview questionnaire consisted of three sections.

**The first section:** was concerned with the socio-demographic characteristics of patients such as age, sex, level of education, job status, and smoking habits.

**The second section included two parts:**

Part 1: It assesses Frequency and severity of symptoms among patients. The frequency symptoms included presence of lost weight, easy fatigability, anorexia, night sweating, fever/rigors, cough, hemoptysis and chest pain during cough. It was developed by the researcher and guided by [5,6]. Frequency of symptoms was self-reported by patients and medical records. To quantify the overall clinical severity of these symptoms, the researcher used the Bandim TB Score [16]. The total severity score is calculated by summing points assigned for specific clinical signs, and the overall symptom severity is classified as follows: Mild (0–2 points), indicating mild weight loss and no dyspnea; Moderate (3–5 points), indicating moderate malnutrition and some dyspnea; and Severe (6 or more points), indicating severe wasting and tachypnea (> 30 breaths/minute). The internal consistency of the symptom severity instrument, as measured by Cronbach's alpha, was 0.75 in the present study. This indicates good reliability and is consistent with the 0.78 coefficient reported in the original validation study [16].

Part 2: adherence to the TB therapeutic regimen was assessed using the 8-item Morisky Medication Adherence Scale (MMAS-8), adopted from [17], which consists of eight questions with "Yes" or "No" responses to categorize patients into three levels: High Adherence (a score of 8), indicating no missed doses; Medium Adherence (a score of 6 to 7), indicating occasional lapses; and Low Adherence (a score of 5 or less), indicating a high risk of non-compliance. *"Formal permission to use the 8-item Morisky Medication Adherence Scale (MMAS-8) was obtained from the copyright holder, Donald E. Morisky. The license number is held on file by the corresponding author and is available upon request."* The internal consistency of MMAS-8 in this study's sample, as measured by Cronbach's alpha, was 0.80 in the present study. This confirms the scale's good reliability and aligns with the 0.83 coefficient reported in its original development study [17].

**The third section:** It assesses patient’s knowledge regarding the disease. It was developed by the researcher and guided by [9,12]. It included a structured questionnaire comprising 14 questions. These questions covered seven key domains: nature of the disease, treatment, compliance, diet regimen, exercise regimen, smoking, and personal hygiene. Each question contained multiple sub-items requiring specific correct responses. A point was awarded for each correct sub-item answer, while incorrect or "don't know" responses received zero points. The total number of correct sub-items across all 14 questions was summed to create a composite (Total Knowledge Score). The maximum possible score was 34 points. To classify overall knowledge, a percentage score was calculated from the total score (Total Score / 34 * 100). Participants were then dichotomized into two groups:

• **Satisfactory Knowledge:** A total percentage score ≥ 60%.

• **Unsatisfactory Knowledge:** A total percentage score < 60%.

The internal consistency of the knowledge questionnaire in this study's sample, as measured by Cronbach's alpha, was 0.76. This indicates an acceptable level of reliability for the developed instrument.

### 4. Data collection

Following an official approval was obtained, the field work was done over eight weeks (June and July 2018), twice a week, on Saturday and Tuesday, from 12 p.m. to 8 p.m. Pre-assessment was done on the first week by obtained information about patients and their prescribed regimen and treatment outcomes from medical records kept by the health services and by the patients. This helped in classifying patients into compliant and non-compliant groups. The researcher interviewed every patient individually. At that time, the nature and purpose of the study were explained to the patient. Written consent for participation was taken, and confidentiality of any obtained information was ensured to the patient. A pilot study was conducted to assess the clarity of the interview questions and estimate the time required to complete the form.

### 5. Data analysis

The data were analyzed using SPSS version 25.0 software (IBM Corp., Armonk, NY, USA). Descriptive statistics were presented as frequencies and percentages for categorical variables and as means and standard deviations for continuous variables. Inferential analyses proceeded in two stages. The independent samples t-test was used to compare the means of continuous variables (e.g., age, body mass index [BMI]) between the two groups, with results reported as (t (degrees of freedom) = [t-value], *p* = [*p*-value]). The Chi-square test (χ^2^) was used for categorical variables, including sociodemographic characteristics, symptom severity, factors affecting adherence, and knowledge levels. Fisher’s exact test was used for contingency tables where more than 20% of the expected cell counts were less than 5. The results for these categorical tests were reported as (χ^2^ (degrees of freedom) = [test value], *p* = [*p*-value]) or (Fisher’s exact test, *p* = [*p*-value]). For all analyses, statistical significance was set at a two-tailed *p*-value of less than .05.

### 6. Ethical considerations

The study was reviewed and approved by the Scientific Research Ethical Committee of the Faculty of Nursing at Ain Shams University (approval code No: 456-4 2021) and then from the director of the ACH in Cairo, Egypt, written informed consent was obtained from all participants or their representatives after explaining the study’s aims, privacy and confidentiality of data were assured for all participants, participants were informed of their right to withdraw from the study at any time without any penalty. The procedures applied in this study were considered safe for all patients.

## RESULTS

### 1. General characteristics of participants

This study compared the socio-demographic characteristics of compliant and non-compliant tuberculous patients. The mean age of the compliant group (42.20 ± 11.70 years) was slightly higher than that of the non-compliant group (37.40 ± 12.00 years), but this difference was not statistically significant. However, statistically significant differences were observed in education, employment status, and BMI. A significantly higher proportion of patients in the compliant group were educated (72.5%) and not working (45.0%), while a significantly lower proportion were weight loss (12.5%). Furthermore, the mean BMI was higher in the compliant group (21.60 ± 2.20) compared to the non-compliant group (20.10 ± 2.70), *p* = .010 (Table 1).

### 2. The frequency and severity of symptoms is compared between patients of the two groups

It is evident that patients in the non-compliant group significantly higher percentages of anorexia, night sweating, fever and rigors, cough, and chest pain during cough. Moreover, about three fourths of them (72.5%) had severe symptoms, compared to 30.0% of the compliant group, *p* < .001 (Table 2).

### 3. The factors affecting adherence to therapeutic regimen of TB among patients in the two groups

The table shows that more compliant patients had enough income for treatment, compared to non-compliant patients, 45.0% and 15.0%, respectively, *p* = .004. Meanwhile, most non-compliant patients (92.5%) sometimes forget to take TB medicine, compared to about half (52.5%) of the compliant, *p* < .001. The assessment of medication adherence using the MMAS-8 revealed a stark and statistically significant disparity in adherence patterns between the groups classified as compliant and non-compliant, *p* < .001.

Within the compliant group, most patients (87.5%) demonstrated medium-to-high adherence. Adherence levels were distributed as follows: High Adherence (score of 8) was observed in 15 patients (37.5%), and Medium Adherence (score of 6-7) was the most common level, found in 20 patients (50.0%). Conversely, only five patients (12.5%) in this group were classified as having Low Adherence (score of ≤ 5).

In stark contrast, the non-compliant group was predominantly characterized by poor adherence. Fully half of the patients in this group (20 patients, 50.0%) were in the Low Adherence category. Only 11 patients (27.5%) showed Medium Adherence, and a mere nine patients (22.5%) achieved High Adherence (Table 3).

### 4. Frequency of satisfactory knowledge about TB

Most of the compliant patients had total satisfactory knowledge about TB (82.5%), compared to about half (52.5%) of the non-compliant group, with a statistically significant difference, *p* =.004. This difference was mostly in knowledge about treatment, which was satisfactory in all compliant patients (100.0%) (Table 4).

### 5. The relation between satisfactory knowledge about TB and patients’ socio-demographic characteristics

In the compliant group, knowledge was significantly affected by age, sex, education, and job status. Thus, younger, male, educated, working patients had more satisfactory knowledge than older, female, illiterate, and not working patients. No such associations could be revealed in the non-compliant group (Table 5).

## DISCUSSION

The study aimed to assess factors affecting treatment compliance and non-compliance among patients with TB. The current study offers important insights into how sociodemographic factors, clinical presentation, knowledge levels, and treatment adherence interact among TB patients in Egypt. Our findings reveal several key patterns that deserve further discussion considering recent evidence from similar settings.

The results confirm a critical educational and economic divide between compliant and non-compliant patients. The high illiteracy rate among non-compliant patients (almost double) presents a fundamental challenge in a context characterized by diverse educational levels. This underscores the need for contextually appropriate health awareness programs that do not rely solely on literacy but utilize simple visual and auditory tools, aligning with the framework proposed by Hargreaves et al. [18]. The financial burden, indicated by most non-compliant patients reporting insufficient income for treatment, reflects the reality of poverty and catastrophic health expenditures in a health system reliant on out-of-pocket payments. This forces patients to choose between working to secure their livelihood and adhering to clinic appointments, a dilemma highlighted by studies in Egypt [5].

The differences in nutritional status confirm that malnutrition increases drug toxicity and decreases treatment tolerance, as established in a clinical review of TB and undernutrition [19]. The significantly higher rate of normal BMI in the compliant group (87.5% vs. 52.5%) and their higher mean BMI (21.6 vs. 20.1) suggest that nutritional status is a critical biological determinant of treatment adherence. This relationship likely operates through a dual pathway: physiologically, adequate nutrition bolsters the immune system and provides the metabolic reserves necessary to tolerate and metabolize powerful TB drugs, reducing the severity of drug-related side effects that often lead to missed doses [10]. Simultaneously, a normal BMI may also be a proxy for better socioeconomic status, reflecting greater household food security and overall stability, which reduces the competing pressures of poverty that force patients to prioritize work or other survival needs over consistent treatment. Therefore, being loss of weight is not merely a symptom of TB but an active barrier to its cure, creating a vicious cycle where the disease worsens malnutrition, which in turn undermines the capacity to complete treatment [17].

BMI is not just an indicator, but a cornerstone of treatment success. The significant gap in BMI between the two groups emphasizes that malnutrition, prevalent in Egypt due to economic and dietary cultural factors, creates a vicious cycle: TB causes wasting, which weakens the body's ability to tolerate drugs and increases their toxicity [19], leading the patient to abandon treatment, which in turn worsens the disease and malnutrition. Therefore, integrating nutritional support into the TB treatment package is not a luxury, but a necessary investment in treatment efficacy, especially for the poorest segments of the population.

Furthermore, analysis of symptom severity shows significant differences in clinical profiles between the groups. Non-compliant patients faced more severe symptoms across various domains, a pattern quantified using a tool like the symptom-based scoring system in a community-based study in Guinea-Bissau [20]. The higher rates of anorexia, fever, and overall severe symptoms in non-compliant patients suggest a potential cycle where more severe symptoms lead to worse adherence. This fits with the "symptom-adherence paradox," where patients with more severe symptoms exhibit poorer adherence due to complex psychosocial factors, a dynamic noted in studies of TB-related stigma, such as the qualitative research on patient experiences in Egypt [10].

This creates a biologically vicious cycle: severe symptoms like fatigue and anorexia can directly reduce a patient's physical and cognitive capacity to adhere to treatment. Furthermore, the near-universal presence of cough in the non-compliant group may indicate a longer duration of illness or treatment failure, which itself is a consequence of non-adherence, thus creating a feedback loop that worsens health outcomes.

The nearly universal occurrence of cough in non-compliant patients may indicate delayed treatment initiation or treatment failure, as noted in recent TB monitoring reports. Forgetting medication and unmanaged side effects represent a significant behavioral and biological barrier. In an overburdened health system, patients may not receive adequate explanation or proactive support to handle expected drug side effects. This creates fear and avoidance of medication. Therefore, adopting practical strategies like reminder systems (e.g., text messages) and strengthening the Directly Observed Therapy (DOT) program with effective counseling can make a significant difference.

Regarding structural and health system factors, the data revealed significant challenges. While physician communication did not show a marked difference between groups, a stark contrast was observed in financial capacity, with far fewer non-compliant patients reporting sufficient income for treatment. This aligns with findings from a cross-sectional study in Indonesia [21], which identified catastrophic health expenses as a primary cause of TB treatment abandonment. Furthermore, the extreme fatigue reported by most non-compliant patients likely reflects both the disease's progression and the physical strain of managing work and treatment, a challenge highlighted in a qualitative study of Bangladeshi garment workers [22].

The study highlights key differences in factors affecting TB treatment adherence between compliant and non-compliant patients. Physical and behavioural factors emerged as significant barriers, with non-compliant patients significantly more likely to miss doses due to side effects and to forget to take their medication. This finding confirms that medication-related challenges, such as adverse effects and forgetfulness, play a crucial role in treatment adherence, as discussed in research on TB-related stigma and patient experiences [10]. Consequently, implementing better side-effect management protocols and reminder systems, such as mobile alerts or DOT, could significantly improve compliance rates, a strategy supported by the implementation framework [18].

The data revealed that structural barriers outweigh social support. The extreme financial constraint reported by non-compliant patients is a critical health-system and economic barrier, as poverty limits access to transportation, nutrition, and the ability to take time off work for clinic visits. The high rates of missed doses due to side effects indicate a biological and clinical management gap; unmanaged drug toxicity creates a physical aversion to medication. Meanwhile, the fact that social support showed no difference suggests that while culturally important, it is insufficient to overcome these profound structural and clinical obstacles [5].

Economic factors also strongly influenced adherence, with compliant patients more likely to have sufficient income for treatment. This underscores the impact of financial constraints on TB care, as poverty may limit access to transportation, nutrition, or other resources needed for consistent treatment, a finding consistent with the systematic review which identified cost as a major barrier across multiple studies [7]. In contrast, social factors like doctor encouragement and family support showed no significant differences between groups, indicating that while these elements are important, they may not be sufficient to overcome structural barriers like cost and medication challenges. This nuanced finding aligns with a cross-sectional study in Nigeria, which highlighted that clinical and structural factors often outweigh social support in determining treatment success [11].

The disparity in adherence levels further emphasizes the need for multifaceted interventions. Non-compliant patients had substantially higher rates of low adherence, suggesting that a combination of financial support, side-effect mitigation, and patient education is essential. This supports the call for integrated approaches—including subsidies for treatment costs, enhanced counselling on medication management, and community-based support systems—as outlined in the WHO's which champions patient-centred care and social protection to improve outcomes in vulnerable populations [13]. The medication regimen for TB is a more powerful tool for adherence than general disease knowledge. This points to a health-system mechanism where patient education may be too generic. The fact that most of non-compliant patients still possessed this knowledge yet did not adhere demonstrates a cultural and behavioural mechanism: knowledge is necessary but not sufficient, as it cannot compensate for a lack of resources or the presence of overwhelming structural barriers like cost and side effects [6].

The knowledge assessment in the current study uncovers major gaps, especially concerning treatment specifics. These results endorse the implementation of "knowledge-aware" TB programs which customize education according to patients' literacy levels and socioeconomic circumstances, as proposed in the implementation framework [18]. The sex difference in knowledge among compliant patients (where more than half of males versus one third of females showed adequate knowledge) contradicts some earlier findings and requires further investigation, possibly due to cultural issues limiting women's health information access, a dynamic noted in the qualitative study on TB-related stigma experienced by women in India [9].

The stark link between female sex and unsatisfactory knowledge in the compliant group reveals a cultural and gender-related barriers, where women may have less access to health information, lower general literacy, or less autonomy to engage with the healthcare system. The strong association between education and knowledge underscores a structural mechanism: health literacy is often dependent on general literacy. An educated individual is better equipped to navigate the health system and understand complex instructions, indicating that public education is a fundamental social determinant of health [23,24].

## CONCLUSION

The finding revealed that compliance was higher among educated working and normal BMI patients. Most of the non-compliant patients had severe symptoms of TB. The factors that might lead to non-compliance were insufficient income for treatment, absence of somebody at home who helps in regulating treatment, decreased motivation for improved health as well as the lack of knowledge regarding diet regimen, smoking, and personal hygiene. These findings compel a paradigm shift in care towards integrated, biologically and socially informed interventions. For clinical nursing, nurses should provide anticipatory guidance, preparing patients for common side effects, and co-creating a simple, actionable management plan with patient-specific adherence counseling. This empowers patients and prevents treatment interruptions that lead to failure or drug resistance.

Concurrently, community health programming must transcend generic education by delivering "knowledge-aware" literacy-appropriate messaging and forging partnerships to mitigate structural barriers like transportation and treatment costs, thereby ensuring that vulnerable patients are supported by both the clinic and the community to successfully complete their treatment. In addition, development and use of low-literacy, high-impact tools. This includes visual aids, pictograms, and audio messages that convey practical, treatment-specific information. The nursing profession can directly dismantle the complex barriers to TB treatment adherence, dramatically improving patient outcomes and advancing public health goals in Egypt.

This study has several limitations that should be considered when interpreting the findings. First, the cross-sectional design captures data at a single point in time, which establishes associations but cannot determine causality between factors like knowledge, symptoms, and adherence. Second, the use of a single setting ACH and a relatively small sample size (n = 80) limits the generalizability of the results to the broader population of TB patients in Egypt or other contexts to validate and extend these findings. Third, the reliance on self-reported data for adherence, symptoms, and some socioeconomic factors is susceptible to social desirability bias and recall error, potentially leading to an overestimation of adherence and knowledge scores.

The future research recommends implementing integrated patient-centered interventions that combine tailored education on treatment management and side-effects with robust social support systems, such as DOT and community health workers, to address knowledge gaps and provide practical adherence assistance.

## Figures and Tables

**Table 1.** Socio-demographic Characteristics of Patients in the Compliant and Non-compliant Groups (N = 40 in Each Group)

Personal characteristics	Compliant	Non-compliant	χ^2^ or t (df)	*p*
Age (years)				
< 40	16 (40.0)	24 (60.0)	χ^2^ (1) = 3.20	.070
≥ 40	24 (60.0)	16 (40.0)		
	42.20 ± 11.70	37.40 ± 12.00	t (78) = 1.82	.070
Sex				
Men	22 (55.0)	26 (65.0)	χ^2^ (1) = 0.83	.360
Women	18 (45.0)	14 (35.0)		
Education				
Illiterate	11 (27.5)	20 (50.0)	χ^2^ (1) = 4.27	.040*
Educated	29 (72.5)	20 (50.0)		
Job status				
Not employed	18 (45.0)	8 (20.0)	χ^2^ (1) = 5.70	.020*
Employed	22 (55.0)	32 (80.0)		
BMI				
Underweight	5 (12.5)	19 (47.5)	χ² (1) = 11.67	< .001*
Normal	35 (87.5)	21 (52.5)		
	21.60 ± 2.20	20.10 ± 2.70	t (78)= 2.71	.010*

Values are presented as the mean ± standard deviation or n (%). df = Degrees of freedom; BMI = Body mass index. *Statistically significant at *p* < .050

**Table 2.** Frequency and Severity of Symptoms among Patients in the Compliant and Non-compliant Groups (N = 40 in Each Group)

Items	Compliant	Non-compliant	F or χ^2^ (df)	*p*
Lost weight	35 (87.5)	36 (90.0)	F	1.000
Easy fatigability	33 (82.5)	37 (92.5)	χ^2^ (1) = 1.83	.180
Anorexia	27 (67.5)	37 (92.5)	χ^2^ (1) = 7.81	.005*
Night sweating	17 (42.5)	26 (65.0)	χ^2^ (1) = 4.07	.040*
Fever/rigors	17 (42.5)	27 (67.5)	χ^2^ (1) = 5.05	.020*
Cough	22 (55.0)	37 (92.5)	χ^2^ (1) = 14.53	< .001*
Hemoptysis	22 (55.0)	27 (67.5)	χ^2^(1) = 1.32	.250
Chest pain during cough	18 (45.0)	28 (70.0)	χ^2^ (1) = 5.12	.020*
Severity of symptoms				
Mild	8 (20.0)	2 (5.0)	χ^2^ (2) = 14.82	< .001*
Moderate	20 (50.0)	9 (22.5)		
Severe	12 (30.0)	29 (72.5)		

Values are presented as n (%). df = Degrees of freedom. *Statistically significant at *p* < .050.

**Table 3.** Factors Affecting Adherence to the Therapeutic Regimen for Tuberculosis among Patients in the Compliant and Non-compliant Groups (N = 40 in Each Group)

Factor items	Compliant	Non-compliant	χ^2^ (df)	*p*
Physical factors				
Treatment started at diagnosis	22 (55.0)	25 (62.5)	0.45 (1)	.500
Has missed doses due to side effects	15 (37.5)	29 (72.5)	9.80 (1)	.002*
Social factors				
Doctor encourages regular attendance	27 (67.5)	31 (77.5)	1.00 (1)	.320
Somebody at home helps in regulating treatment	22 (55.0)	18 (45.0)	0.80 (1)	.370
Behavioral factors				
Has motivations for improved health	25 (62.5)	19 (47.5)	1.82 (1)	.180
Sometimes forgets to take TB medicine	21 (52.5)	37 (92.5)	16.05 (1)	< .001*
Economic factors				
Enough income for treatment	18 (45.0)	6 (15.0)	8.57 (1)	.004*
Doctor answers your questions	26 (65.0)	24 (60.0)	0.21 (1)	.640
Adherence levels				
High	15 (37.5)	9 (22.5)	13.12 (2)	.001*
Moderate	20 (50.0)	11 (27.5)		
Low	5 (12.5)	20 (50.0)		

Values are presented as n (%). df = Degrees of freedom; TB = Tuberculosis. *Statistically significant at *p* < .050.

**Table 4.** Frequency of Satisfactory Knowledge about Tuberculosis among Patients in the Compliant and Non-compliant Groups (N = 40 in Each Group)

Satisfactory knowledge about	Compliant	Non-compliant	χ^2^ (df)	*p*
Nature of disease	27 (67.5)	33 (82.5)	2.40 (1)	.120
Treatment	40 (100.0)	29 (72.5)	12.75 (1)	< .001*
Compliance	32 (80.0)	25 (62.5)	2.99 (1)	.080
Diet regimen	33 (82.5)	30 (75.0)	0.67 (1)	.410
Exercise regimen	12 (30.0)	10 (25.0)	0.25 (1)	.620
Smoking	27 (67.5)	26 (65.0)	0.06 (1)	.810
Personal hygiene	29 (72.5)	26 (65.0)	0.52 (1)	.470
Total knowledge	33 (82.5)	21 (52.5)	8.21 (1)	.004*

Values are presented as n (%). df = Degrees of freedom. *Statistically significant at *p* < .050.

**Table 5.** Relation between Satisfactory Knowledge about Tuberculosis and Patients’ Socio-demographic Characteristics

Variables	Knowledge in Compliant			Knowledge in Non-compliant		
	Satisfactory	Unsatisfactory	Test (*p*)	Satisfactory	Unsatisfactory	Test (*p*)
Age (years)						
< 40	16 (48.5)	0 (0.0)	Fisher's Exact	12 (57.1)	12 (63.2)	X^2 ^= 0.15, df = 1
≥ 40	17 (51.5)	7 (100.0)	.030*	9 (42.9)	7 (36.8)	.700
Sex						
Men	22 (66.7)	0 (0.0)	Fisher's Exact	16 (76.2)	10 (52.6)	X^2^ = 2.43, df = 1
Women	11 (33.3)	7 (100.0)	< .001*	5 (23.8)	9 (47.4)	.120
Education						
Illiterate	6 (18.2)	5 (71.4)	Fisher's Exact	11 (52.4)	9 (47.4)	X^2 ^= 0.10, df = 1
Educated	27 (81.8)	2 (28.6)	.010*	10 (47.6)	10 (52.6)	.750
Job status						
Not employed	12 (36.4)	6 (85.7)	Fisher's Exact	4 (19.0)	4 (21.1)	Fisher's Exact
Employed	21 (63.6)	1 (14.3)	.030*	17 (81.0)	15 (78.9)	1.00

Values are presented as n (%). df = Degrees of freedom. *Statistically significant at *p* < .050.

## Data Availability

The dataset supporting the conclusions is available from the corresponding author on reasonable request.
